# Aquatic macrophyte dynamics in the Danube Inland Delta over the past two decades: homogenisation or differentiation of taxonomic and functional community composition?

**DOI:** 10.1007/s10661-025-13777-1

**Published:** 2025-02-27

**Authors:** Pavel Beracko, Silvia Kubalová, Igor Matečný

**Affiliations:** 1 Department of Ecology, Faculty of Natural Sciences, Comenius University in Bratislava, Bratislava, Slovakia; 2 Department of Botany, Faculty of Natural Sciences, Comenius University in Bratislava, Bratislava, Slovakia; 3 Department of Physical Geography and Geoinformatics, Faculty of Natural Sciences, Comenius University in Bratislava, Bratislava, Slovakia

**Keywords:** Long-term monitoring, Lowland river, Aquatic plants, Diversity, Species traits

## Abstract

Our study provides insights into the spatio-temporal dynamics of macrophyte assemblages in the Danube Inland Delta, situated in the northwest part of the Pannonian lowland on the Slovak-Hungarian border. A total of 63 aquatic plant species were recorded at six monitored sites during the years 2003–2020, including 1 endangered, 2 vulnerable, and 1 neophyte species. Macrophyte data from long-term monitoring were used to test the hypothesis that aquatic plant assemblages in the hydrologically disturbed Danube riverscape have become more taxonomically and functionally similar over the past 17 years. Although a decline in the taxonomic and functional richness of macrophyte assemblages was observed at most monitoring sites, no evident year-to-year decrease in their heterogeneity was noted for either aspect of biodiversity. However, a significant decline in taxonomic and functional richness, as well as in the heterogeneity of the macrophyte community across the entire monitored area, was observed during the study period. While a decrease in species number represented approximately one species per year, the taxonomical beta diversity, including its taxonomical turnover component, also declined, with a mean annual decrease of 0.5% and 0.7%, respectively. Using abundance class data, the mean annual decrease in beta diversity was 0.6%. A time-series analysis of macrophyte functional traits (growth form, dispersal unit, and strategy) revealed that aquatic plant assemblages from different monitoring sites converged toward a narrower range of functional structure. The mean annual decline in macrophyte functional heterogeneity was 0.7% based on abundance class data and approximately 0.3% based on incidence data.

**Supplementary Information:**

The online version contains supplementary material available at 10.1007/s10661-025-13777-1.

## Introduction

The Danube, with its tributaries, represents one of the largest river systems in Europe, which either directly or indirectly affects the lives of 80 million people (Atanackovič et al., 2020). It is also home to more than 2000 plant and around 5000 animal species, including numerous endangered and several nearly extinct taxa. As one of the European biodiversity hotspots, the Danube watercourse interconnects different bioregions across Europe and, therefore, creates important bio-corridors between them. From this viewpoint, joint international monitoring surveys of the Danube waters (e.g. JDS1–JDS4) are crucial for the correct assessment of the changes in river environment quality and ecological status across countries of the Danube region (Mănoiu & Crăciu, 2021). Moreover, these transnational approaches also provide effective settings and harmonisation of national monitoring practices and procedures for the development of the international strategy for the protection of the waters in the Danube catchment area based on the UNECE Convention on the Protection and Use of Transboundary Waters (Helsinki Convention). After the acceptance and implementation of transnational rules in monitoring and quality assessment of aquatic biotopes, the local monitoring programs of Danube waters can be an important complement to the global national and international monitoring activities. Moreover, they are usually easily manageable and often assess changes in the quality of riverine biotopes and biodiversity at much finer spatio-temporal scales. For this reason, local monitoring programs are crucial not only for the local but also for the joint international approach to the management of Danube biodiversity conservation (Tittizer & Banning, 2001; Zavadsky & Liska, 2015).

Long-term data and studies based on them are necessary for identifying natural and anthropogenic-induced alterations of the riverine landscape and documenting spatio-temporal changes in the communities and functional interactions within river ecosystems (Naiman et al., 1995). Therefore, long-term data generally form a background for understanding the present state of the riverine landscape, as well as the effect of regional and local processes (e.g. extirpation of native species and the introduction and expansion of non-native and invasive species) on biodiversity and ecosystem function losses at different scales (Bai et al., 2004; Sukhotin & Berger, 2013; Tilman et al., 2006).

The regional species pool of a plant community can be changed through the incoming species from communities of other regions or by species extinction within a region (Puhl et al., 2014). In this context, the local species richness strongly depends on the structure of the regional species pool, which determines its potential availability for recovery of locally disturbed communities or colonisation of new suitable habitats (Stohlgren et al., 2008). Thus, the range of structural change in a community on a regional scale, i.e. dynamics at the metacommunity level, is determined through the equilibrium between the rates of within-site species extinction and new patches colonisation (MacArthur & Wilson, 1967). These rates are mainly influenced by the size and regime of disturbances, as well as local biotic interactions. On the one hand, the extinction rates are altered by the magnitude and intensity of environmental stressors and also species’ competitive exclusion. On the other hand, the colonisation rate is given by the input of propagules of indigenous or new species, as well as the presence and availability of regeneration patches characterised, e.g., by moderate disturbance or a low quantity of suitable resources (Booker et al., 2015; Merritt et al., 2010). As both above-mentioned processes usually co-occur in nature, they fully define the range of community dynamics at local and regional scales (Wardle et al., 2011; Tatsumi et al., 2021). Changes in diversity, as the basic feature of the community, can happen over time on one or both scales (Caley & Schluter, 1997; Sax & Gaines, 2003; Thomas, 2013; Vellend et al., 2013). When the regional richness does not change, the increase of local (alpha, within-site) diversity is at the expense of a decrease in between-site (beta, species turnover) diversity, a process defined as biotic homogenisation (Olden & Rooney, 2006). Communities where compositional turnover (simultaneously with natural mechanisms of the species input and extinction) is maintained without local and regional richness changes are considered species-saturated (Elmendorf & Harrison, 2011). If the ecosystem is not species-saturated, then regional and local richness has the potential to increase, a process known as biotic differentiation (Blowes et al., 2024; Sax & Gaines, 2008).

Understanding the rules governing community assembly processes is a cornerstone of modern ecology, providing insight into community dynamics and their underlying mechanisms (Weiher & Keddy, 1999). Current perspectives on community assembly are framed by two theories. The first is the classic view, which posits that community assembly is driven by niche-related processes, such as environmental filtering and species interactions (Tilman, 1982). The alternative view suggests that community assembly is largely governed by neutral processes, where species are ecologically equivalent, and stochastic events dominate (Hubbell, 2001). Niche-related processes (Cavender-Bares et al., 2009) involve selection pressures from environmental gradients and biotic interactions, including competition, facilitation, mutualism, and predation. In contrast, neutral processes (Hubbell, 2001) encompass stochastic factors such as random disturbances, probabilistic dispersal, and birth–death events (Hanson et al., 2012).

In natural ecosystems, the intensity of regional-scale disturbance can characterise the consequences of local colonisations and extinctions for beta diversity (Caswell & Cohen, 1991). Moderate disturbance can create a mosaic of various habitats and potentially incite higher between-habitat biodiversity, while higher and extensive disturbance can reduce the regional environment heterogeneity and lead so to metacommunity homogenisation (Gustafsson et al., 2012; Kouki & Salo, 2020). In this context, biotic homogenisation can represent a potentially multifaceted process that encompasses the loss of not only taxonomic but also genetic and functional distinctiveness over space and time (Olden et al., 2004). As mentioned, this process is driven through two native ecological mechanisms, i.e. species colonisation/invasion and extirpation, and the range and manner in which both mechanisms occur in the environment may define very different levels of homogenisation or differentiation of the community (Olden & Poff, 2003). The impact of both on the community dynamics and ecosystem function is increasingly evaluated through changes in functional group composition (Wardle et al., 2011). This fact suggests that the view through functional heterogeneity of plant community may be more informative for the assessment of ecological integrity than the taxonomic approach, as it reflects resource dynamics and whole ecosystem balance (Loreau, 2000; Pokorný et al., 2005). Functional diversity refers so to the traits of organisms that determine their performance in an environment and their impact on ecosystem functioning. It offers valuable insights into species-environment interactions. Unlike taxonomic diversity, which emphasises species identity, functional diversity focuses on unique characteristics of biotic assemblages and ecosystems, providing a complementary perspective. Together, these approaches capture ecological differences at the community level, whether related to function, niche, or evolutionary history (Cadotte et al., 2013). At the level of plant functional traits, interspecific competition within a local community can exclude ecologically similar species, leading to trait divergence (Herben & Goldberg, 2014). This mechanism is most prevalent in stable and benign environments (Magalhaes & Bernasconi, 2014). Conversely, environmental filtering drives the convergence of trait patterns (Cornwell et al., 2006), a process typical of ecologically unstable habitats (Weiher et al., 1998). Functional traits related to life-strategy attributes, such as growing season and types of growth forms, limit the timing of species establishment and possible competitive interactions (Byun et al., 2013). Therefore, the global understanding of macrophyte community dynamics is simultaneous with registered taxonomic changes, also conditioned by analysing changes in the proportion of functional groups based on relevant species traits. Moreover, the knowledge of the structural and functional attributes of the macrophytes is also crucial for the assessment of the ecological status according to the Water Framework Directive (WFD, 2000/60/EC).

In the last three decades, the state of aquatic macrophyte vegetation has been extensively explored and discussed in various countries of the Danube region (e.g. Pall et al., 1996; Janauer & Wychera, 2000; Janauer & Steták, 2003; Rath et al., 2003; Vukov et al., 2006; Schütz et al., 2006; Ot’ahel’ová et al., 2007; Gyosheva et al., 2020; Janauer et al., 2021). Research in the Lower Danube has documented a decrease in species richness and a structure simplification of submerged vegetation due to eutrophication over two decades since the 1980s (Cristofor et al., 2003). These processes included a restructuring of primary producers, characterised by the suppression of aquatic weeds in some lakes and a reduction in species richness to a few populations dominated by upright and floating growth strategies, especially *Ceratophyllum demersum* and *Potamogeton* species.

In contrast, in the middle Danube reaches, an increase in hydrophyte species richness was observed a few years after the Gabčíkovo Hydropower Plant (GHP) went into operation (Rath, 1997; Oťaheľová & Valachovič, 2002; Rath et al., 2003). Since 2005, scientific papers evaluating changes in aquatic flora in the area affected by the GHP have been absent. The results of their every-year monitoring were only summarised in annual reports, which were elaborated according to the agreement between the Slovak and Hungarian Republic regarding the joined Slovak-Hungarian monitoring of the natural environment and biota. Therefore, we decided to use the macrophyte database acquired from this long-term monitoring and analyse changes in aquatic macrophyte vegetation during the 17 years (from 2003 to 2020). This study aimed to (i) define changes in taxonomical structure and richness macrophytes at the particular monitoring sites, as well as at the whole monitored area, during almost two decades, and (ii) characterise changes in functional composition and richness aquatic vegetation also within monitoring sites and the whole monitored area. We hypothesised that aquatic plant assemblages in the hydrologically disturbed Danube riverscape have become more taxonomically and functionally similar over the past 17 years, especially at the monitoring sites within the bypass section of the Danube, as well as in the whole monitored area. Our assumption of this homogenisation is based on the fact that artificial spring and summer floods have been absent in the area of the GHP bypass section over the past two decades. These artificial floods were intended to substitute natural flooding in the Danube inland delta, which was disturbed by the significant discharge reduction (approximately $\frac{1}{5}$ of the original discharge) after the GHP went into operation at the end of 1992. Until 2001, these simulated floodings had been regular, ensuring at least a short-term lateral and longitudinal connection of particular riverine habitats.

## Methods

### Description of study area and sites

The area covering all monitored sites is situated in the central part of the intermountain depression (between the Alps and the Carpathians), the Danube basin, which is named in Slovakia “Podunajská nížina” (Danubian Lowland). The geological boundary (transition between granite and andesite bedrock) and tectonic subsidence of the basin determine the decrease of the surface slope and slowing down the Danube current velocity in this area. These attributes allowed the development of the specific riverine landscape there, the so-called Danube Inland Delta. It is an alluvial fan located below the granite threshold under Bratislava (between rkm 1799 and 1856). Geographically, the Danube Inland Delta is located within the Szigetköz region in northwestern Hungary and the adjacent areas in Slovakia. It has the typical hydromorphology of a lowland river system, i.e. branching of the main channel, occurrence of lotic and semi-lotic side arms, oxbow lakes and marshlands, presence of meanders, coarse sediment accumulation, etc.

**Fig. 1:**
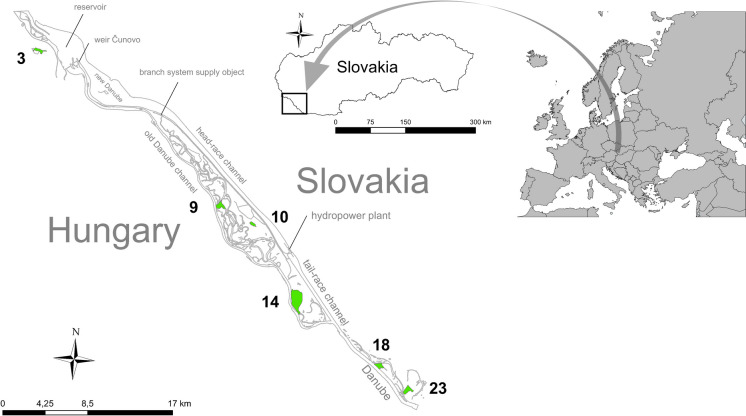
Map of the Gabčíkovo barrage system with monitored sites indicated by green colour

Before embankment and endikement were realised at the end of the nineteenth and at the beginning of the twentieth century, the Danube under Bratislava was a free-flowing braided river (without a main channel) with a wide floodplain. After building up flood protection dikes on both sides, the main Danube channel was created across previous meanders. Consequently, the flow was concentrated into the main channel, and interconnection and interaction between side arms were reduced. In October 1992, the Danube Inland Delta between Rusovce and Gabčíkovo (rkm 1856–1820) had undergone significant hydrological changes due to the operation of the GHP. The GHP has bypassed the old main channel, and its floodplain side arms (Fig. 1). The discharge decrease in the old main channel (approximately $\frac{1}{5}$ of the original discharge—ca. 2000 m^3^ s^−1^) has disrupted the active connection within the arms system, significantly altering the hydrological dynamics in this floodplain area. This impact was partially mitigated by spring and summer simulated floods realised through the artificial supplied system in Dobrohošť until 2001. Since this period, partial connecting between riverine habitats has been performed by increased water inflow into the inland Danube delta through water supply objects during higher discharge in the Danube (over 3500 m^3^ s^−1^ at the Bratislava-Devin station). Monitored water bodies represent two types of floodplain river habitat, i.e. semi-lotic (open) and lentic (separated) oxbows. A short description of the monitoring localities and plots is as follows:

*Monitoring site MP3 (Ostrovné lúčky)* is situated on the dead arm in the area of the Čunovo reservoir downstream from Bratislava. Under the pre-dam condition, the arm was dried up and covered by a softwood floodplain forest. After the GHP was put into operation, the groundwater level increased considerably (by 2–4 m) compared to the pre-dam state. Since 1996, the stagnant water has remained in the willow stand in the former arm bottom. Since 1997, the surface water level has stabilised at 30–50 cm. The monitoring plot was situated on the eastern side of this arm, with a length of 100 m.

Monitoring sites MP9 (Bodícka brána), MP10 (Kráľovská lúka), and MP14 (Istragov) are situated in the within-dike zone between the bypass canal and the old Danube riverbed. The GHP’s influence has manifested itself in a decline in ground and surface water levels and changes in the flood regime.

*Monitoring site MP9 (Bodícka brána)* is situated at the northern edge of the Šulianske rameno arm. It is a deep semi-lotic arm, where the relatively stable water level has been maintained throughout the year. The considerable fast streaming and depth do not create optimal conditions for the many macrophyte species there. The length of the monitored plot there was 100 m.

*Monitoring site MP10 (Kráľovská lúka)* represents the dead arm with the status of protected area. It is situated immediately at the foot of the dike. The eastern part is shallower than the western. Its shores are bordered by a reed stand, except the side along the dike. At this site, three plots were monitored with lengths of 20 m, 300 m, and 200 m.

*Monitoring site MP14 (Istragov island)* is located in the island’s peripheral arm. Its northern part is divided into two branches, and the monitoring was performed on the southern branch. This arm branch is at a considerably advanced stage of terrestrialisation and rarely flooded. Several wooden species penetrating the shrub layer confirm this trend. Three plots were monitored there with lengths of 150 m, 50 m, and 20 m.

The monitoring sites MP18 (Sporná sihoť) and MP23 (Starý les) are also located in the within-dike zone, downstream of the confluence of the tailrace canal with the old Danube channel.

*Monitoring site MP18 (Sporná sihoť)* covers one of the less cut-off branches of the Opátske rameno arm and a small side arm. The water level fluctuation directly depends on the water level in the Danube. At this site, three plots were monitored with lengths of 150 m, 20 m, and 50 m.

*Monitoring site MP23 (Starý les)* is in the Danube stretch, which is not directly influenced by the GHP operation. It is situated in the relic river oxbow immediately at the foot of the dike, which is at an advanced degree of terrestrialisation. Three plots were monitored there with lengths of 20 m, 500 m, and 300 m.

### Field sampling and data processing

In the area affected by GHP operation, continual monitoring of the spatio-temporal changes of macrophytes according to the above-mentioned agreement between Slovakia and Hungary started in 1999. Indirect and sporadic information about aquatic and wetland vegetation from 1991 to 1998 can be only found in some papers or annual reports (e.g. Hodálová & Zaliberová, 1995; Králik, 1996; Oťaheľová, 1991a, b; Šomšák, 1999; Šomšák & Kubíček, 1995; Zaliberová, 1991). Since 1999, six sites have been monitored in the GHP-affected stretch of the Danube. Until 2003, the aquatic vegetation was sampled in the sense of the Zürich-Montpellier school (Braun-Blanquet, 1964). Since 2003, another approach has been applied in sampling macrophyte vegetation, namely the Kohler method of mapping macrophytes (Kohler, 1978). This change was implemented to align with Hungarian monitoring of the Danube and other aquatic vegetation programs in Germany, Austria, and other countries, as a standardised field sampling method enables meaningful comparisons of monitoring results across Danube countries.

In short, according to the Kohler method, the water body is divided into sections with relatively homogeneous ecological conditions. Their length is different but should be easily identifiable in the field. In each section, an inventory of the species of aquatic vegetation (hydrophytes) and other life forms bound to the aquatic environment is made, with a semi-quantitative assessment of the occurrence of each species in a five-classes abundance group (PME–Plant Mass Estimates), 1 – rare species, 2 – occasional species, 3 – frequent species, 4 – abundant species, 5 – very abundant species. Plant quantity expresses the three-dimensional mass of plant material of a particular species in relation to its distribution within the examined section, i.e. it is an expression of the amount of biomass.

In this paper, we evaluate changes in macrophyte vegetation at particular monitored sites only from 2003 to 2020, and that is because of using the same field sampling method. Since 2021, temporal and spatial optimisation and the setting of Danube’s monitoring have gradually been realised, which did not allow the last 3 years to be included in the analyses.

The monitoring of aquatic macrophytes was carried out at the selected sites three times per year—seasonally (spring, summer, and autumn) from 1991 to 2020. For each site and year, the data from the seasonal monitoring occasions were expressed as a more frequent abundance class for each species (in the case of different abundance classes for each season, the mean value was used). Species names are unified according to Marhold and Hindák (1998).

### Homogenisation measurement and statistical analysis

The change of alfa and beta diversity was used to analyse the degree of taxonomical and functional homogenisation/differentiation of the assemblages and the whole macrophyte community. Quantifying biodiversity changes is generally based on the spatio-temporal trends in two measures: alpha and beta diversity (Whittaker, 1972). Directional change in alpha and beta diversity refers to the process of either decreasing or increasing dissimilarity among assemblages over time and space (Magurran et al., 2015), referred to as “biotic homogenisation” and “biotic differentiation”, respectively (Olden & Rooney, 2006).

For taxonomic alpha diversity, the commonly used metrics are species richness and Simpson and Shannon diversity. Unlike the other two metrics, species richness is a simple measure that does not require species abundance. The relationships between species richness and year were explored with linear, exponential, logarithmic, and quadratic models. The best model was selected based on the highest value of the coefficient of determination (*R*^2^). To assess the significance of trends in species richness, two-sided randomisation tests based on 9999 permutations were performed. In these tests, the response variable was randomly shuffled to build a null distribution of the regression coefficient, which was then used to evaluate the significance of the observed coefficient.

For the taxonomic beta diversity evaluated on the presence/absence data, the Sørensen dissimilarity (βsor) was used. Sørensen dissimilarity is created from two additive components, i.e. species turnover and nestedness-resultant dissimilarities (βsne). The Simpson dissimilarity (βsim) defines the contribution of species turnover to beta diversity without the influence of richness gradients (Koleff et al., 2003). In case βsor and βsim are equal, no species nestedness is present in the two assemblages. On the other hand, their difference represents a measure of the nestedness-resultant component to beta diversity so that βsne = βsor − βsim (Baselga, 2010). For this reason, the contribution of species turnover to beta diversity was also measured. For the semi-quantity data (based on the abundance classes data), beta diversity was represented by the Bray–Curtis dissimilarity (βbray). The within-site beta diversity (temporal beta diversity) was so expressed as the vector of βsor, βsim, and βbray of the assemblages of the successional years. The between-site beta diversity (spatial beta diversity) was expressed as the matrices of the pairwise dissimilarities (βsor, βsim, βbray) of assemblages for each monitored year. Temporal changes in taxonomical beta diversity of within-site assemblages and the whole macrophyte community were fitted by linear, exponential, logarithmic, and quadratic models. The best-fitted model was selected based on the highest value of the coefficient of determination (*R*^2^).

The comparison of the dynamics of overall taxonomical community composition relative to within-site dynamics of assemblages over time was analysed and visualised through a multivariate statistical technique named principal response curves (PRC). The PRC was performed on the abundance class data. Each year, the reference community was composed of taxa whose abundance was determined as the mean value of their abundance classes at the sites.

The functional alpha diversity of the macrophyte assemblages and the whole community was expressed as their functional richness (FRic, Villéger et al., 2008) and functional dispersion (FDis, Laliberté & Legendre, 2010), which define convex hull volume in trait space and average distance to the trait centroid, respectively. Their calculations were based on the abundance class data matrix. The trait matrix consisted of the type of growth form, dispersal strategy, and dispersal unit (Supplementary material Appendix Table 1). The classification of plants into categories of growth forms was made according to Janauer (2003); for the sake of clarity of the results in the figures and tables, the category “submersed pleustophytes” is abbreviated as “mesopleustophytes”, the category “submersed anchored macrophytic plants” as “rhizophytes”, and the category “floating leaf rhizophytes” as “floating rhizophytes”. Dispersal trait data were extracted from the Ecological Flora database (Fitter & Peat, 1994, available online at http://www.ecoflora.co.uk). The relationships between functional alpha diversity measures and year were explored with linear, exponential, logarithmic, and quadratic models. The best model was selected based on the highest value of the coefficient of determination (*R*^2^). Like in species richness, two-sided randomisation tests based on 9999 permutations were performed to assess the significance of trends in functional diversity.

**Table 1:** Mean alpha diversity and the prediction of the species richness at each site during the monitoring period (2003–2020)

Sites	Mean species number (alpha diversity)	Maximal species number	Minimal species number	Equation of trend line (within-site temporal change in species number)	Coefficient of determination *R*^2^ (%)	Randomisation test for regression coeffcient (permuted *p*-value)
MP3	8.17	11	6	*y* = 344.6 − 0.167 * year	31.49	0.019
MP9	6.17	10	2	*y* = − 110.5 + 0.058 * year	1.83	0.603
MP10	17.35	21	12	*y* = 508.1 − 0.244 * year	27.13	0.033
MP14	13.17	21	8	*y* = 896.9 − 0.439 * year	36.8	0.009
MP18	20.4	31	13	*y* = 1239.2 − 0.606 * year	48.2	0.002
MP23	14.52	21	6	*y* = 1179.8 − 0.579 * year	54.1	< 0.001
Whole area	41.65	54	28	*y* = 2273.2 − 1.109 * year	62.6	< 0.001

The within-site functional beta diversity was expressed as the vector of Bray–Curtis dissimilarities (used for abundance class data in year x species matrix) and Sørensen dissimilarities (used for incidence data in year x species matrix) of the assemblages’ functional composition of successional years. For each site, the matrix of assemblages’ functional composition was built by multiplying the year x species matrix with the species x trait matrix. The between-site functional beta diversity was expressed as the matrix of pairwise Bray–Curtis dissimilarities (used for abundance class data in year x species matrix) and Sørensen dissimilarities (used for incidence data in year x species matrix) of the assemblages’ functional composition. For each year, the matrix of assemblages’ functional composition was built by multiplying the site x species matrix with the species x trait matrix. Temporal changes in functional beta diversity of within-site assemblages and the whole macrophyte community were fitted by linear, exponential, logarithmic, and quadratic models. The coefficient of determination (*R*^2^) was used to select the best-fitted model.

Similar to taxonomical data, the dynamics of overall functional community composition relative to within-site dynamics of assemblages over time were compared and plotted through the PRC. The matrix of assemblages’ functional composition was constructed from the abundance class data matrix and species trait matrix. Each year, the reference community was composed of taxa whose abundance was determined as the mean value of their abundance classes at the sites.

All calculations were made in R 4.2.1 (R Core Development Team, 2022) by using the vegan (Oksanen et al., 2022), FD (Laliberté et al., 2014) and ade4 (Dray & Dufour, 2007) packages.

## Results

### Macrophyte taxonomic diversity and its change

In total, 63 aquatic macrophyte taxa (including 1 endangered, 2 vulnerable, and 1 neophyte species) were found at six monitoring sites within the Danube Inland Delta (between Danube rkm 1799 and 1856). Higher species richness generally occurred in the downstream sites of the monitored area (Table 1). Species *Carex acuta/gracilis*, *Ceratophyllum demersum*, *Iris pseudacorus*, *Lemna minor*, *Phragmites australis*, *Rorippa amphibia*, and *Spirodela polyrhiza* belonged to the most frequent in the spatio-temporal range of the whole community, which were present in more than 50% of the assemblages.

At all sites (except MP9) and the whole area, a significant decrease (*p*-value < 0.05) in the species richness was observed from 2003 to 2020. At the MP9, only a slight increase in species richness happened, which is documented by the very low positive regression coefficient and prediction ability of the model (Table 1). Based on the regression coefficient, the highest decline in species number was found at the downstream sites (MP14–MP23). In the monitored area, the mean annual decrease of species richness was approximately one species per year.

**Fig. 2:**
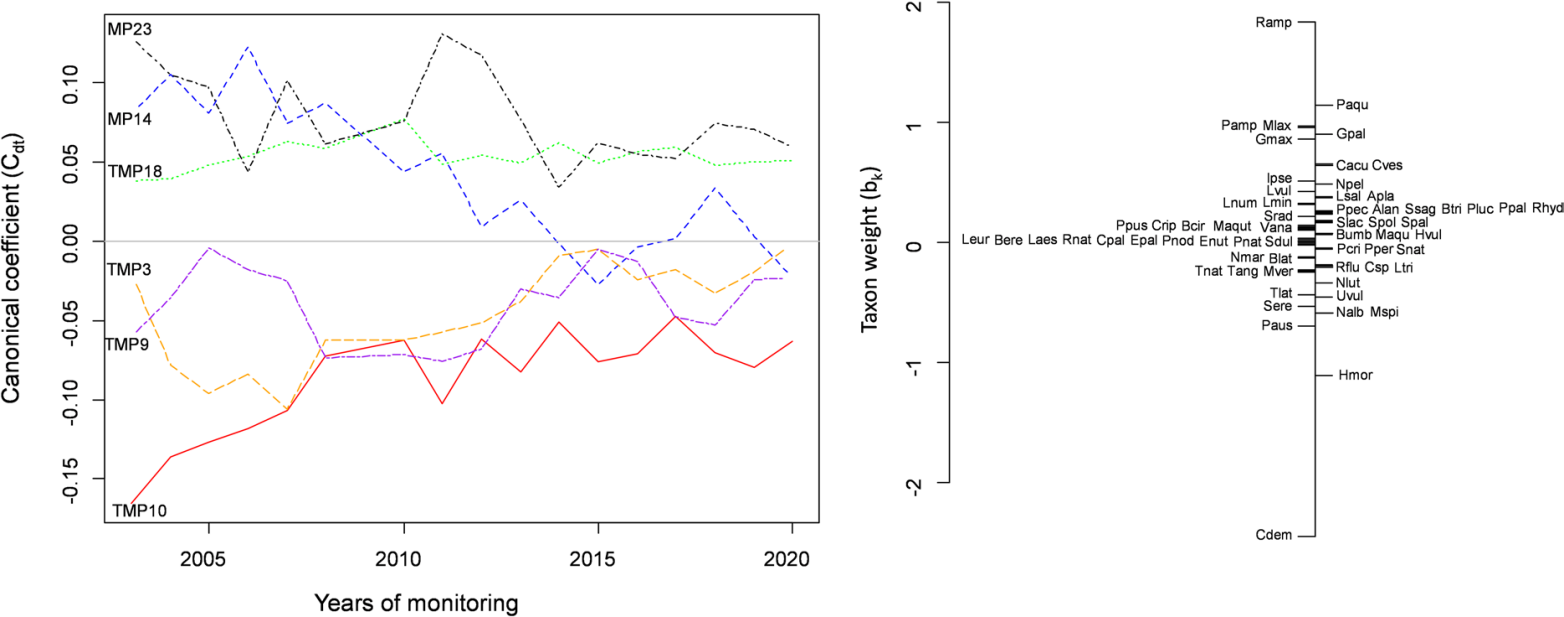
Principal response curves (PRC) showing temporal changes in the structure of the macrophyte assemblages at the monitoring sites. The vertical axes (left and right) represent the first canonical axis of the Redundancy Analysis conducted on the species abundance classes of particular macrophyte species. Macrophyte species abbreviations: Alan—*Alisma lanceolatum*, Apla—*Alisma plantago-aquatica*, Bcir—*Batrachium circinatum*, Btri—*Batrachium trichophyllum*, Bere—*Berula erecta*, Blat—*Bolboschoenus laticarpus*, Bumb—*Butomus umbellatus*, Cpal—*Callitriche palustris*, Cacu—*Carex acuta/gracilis*, Crip—*Carex riparia*, Cves—*Carex vesicaria*, Cdem—*Ceratophyllum demersum*, Enut—*Elodea nuttallii*, Epal—*Equisetum palustre*, Gpal—*Galium palustre*, Gmax—*Glyceria maxima*, Hvul—*Hippuris vulgaris*, Hmor—*Hydrocharis morsus-ranae*, Ipse—*Iris pseudacorus*, Lmin—*Lemna minor*, Ltri—*Lemna trisulca*, Laes—*Leucojum aestivum*, Leur—*Lycopus europaeus*, Lvul—*Lysimachia vulgaris*, Lnum—*Lysimachia nummularia*, Lsal—*Lythrum salicaria*, Maqu—*Mentha aquatica*, Mlax—*Myosotis laxiflora*, Maqut—*Myosoton aquaticum*, Mspi—*Myriophyllum spicatum*, Mver—*Myriophyllum verticillatum*, Nmar—*Najas marina*, Nlut—*Nuphar lutea*, Nalb—*Nymphaea alba*, Npel—*Nymphoides peltata*, Pamp—*Persicaria amphibia (Polygonum amphibium)*, Paqu—*Phellandrium aquaticum (Oenanthe aquatica)*, Paus—*Phragmites australis*, Ppal—*Poa palustris*, Pcri—*Potamogeton crispus*, Pluc—*Potamogeton lucens*, Pnat—*Potamogeton natans*, Pnod—*Potamogeton nodusus*, Ppec—*Potamogeton pectinatus*, Pper—*Potamogeton perfoliatus*, Ppus—*Potamogeton pusillus*, Rflu—*Riccia fluitans*, Rnat—*Ricciocarpos natans*, Ramp—*Rorippa amphibia*, Rhyd—*Rumex hydrolapathum*, Ssag—*Sagittaria sagittifolia*, Snat—*Salvinia natans*, Slac—*Schoenoplectus lacustris*, Srad—*Scirpus radicans*, Sdul—*Solanum dulcamara*, Sere—*Sparganium erectum*, Spol—*Spirodela polyrhiza*, Spal—*Stachys palustris*, Tnat—*Trapa natans*, Tang—*Typha angustifolia*, Tlat—*Typha latifolia*, Uvul—*Utricularia vulgaris*, Vana—*Veronica anagallis-aquatica*

The PRC analysis result showed that 87.7% of total variation in species composition was explained by within-site variation (including its interaction with year), whereas 12.3% was explained by between-year variation, 22.1% of which is displayed in the diagram. From the PRC diagram, an evident increase in the taxonomical similarity of the macrophyte community is observed during its 20-year monitoring (Fig. 2).

In the PRC analysis, the multiple of b_k_ × C_dt_ (signed as T_dtk_) determines the response pattern of each taxon in the community over time. In more detail, C_dt_ represents the canonical coefficient of site effect (d) at time (t), quantifying the community response pattern relative to the mean community along the first canonical axis of the partial RDA. Similarly, b_k_ denotes the species-specific multiplier of the canonical coefficient, meaning that T_dtk_ (b_k_ × C_dt_) describes the response pattern of species k expressed relative to its mean abundance. In the upstream sites (MP3–MP10); therefore, the most affected macrophyte taxa were *C. demersum* and *Hydrocharis morsus-ranae*, both with negative weights, indicating a successional decline in their abundance during the monitored period. In the downstream sites (MP14–MP23), the most affected macrophyte taxa were *R. amphibia*, *Phellandrium aquaticum*, *Persicaria amphibia*, *Myosotis laxiflora*, *Galium palustre*, and *Glyceria maxima*, all with positive weights, also indicating a successional decline in their abundance during the monitored period.

**Fig. 3:**
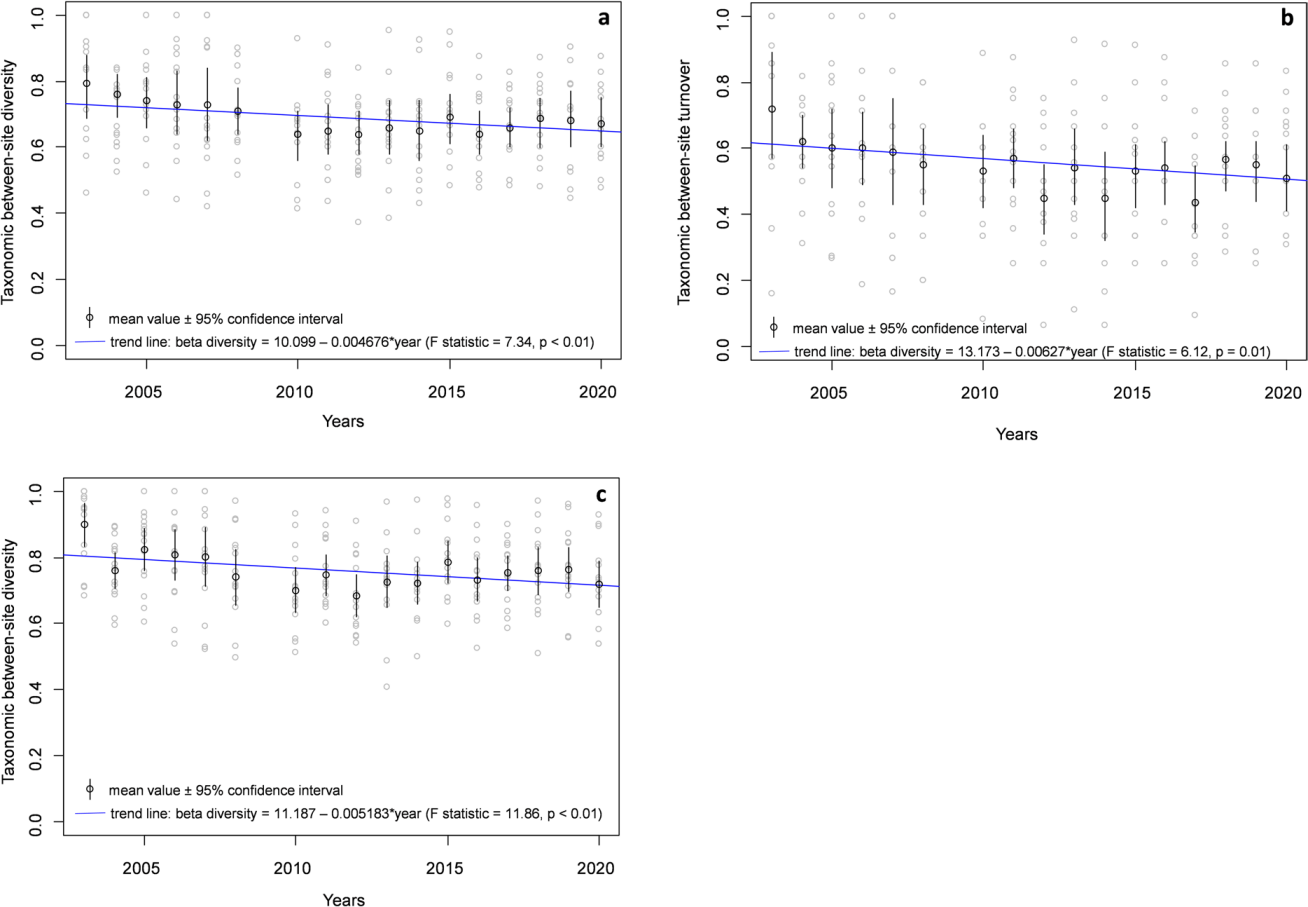
Change of the taxonomic beta diversity (Sorenson pairwise dissimilarity based on presence/absence data—**a**), taxonomic turnover (Simpson pairwise dissimilarity based on presence/absence data—**b**), taxonomic beta diversity (Bray–Curtis pairwise dissimilarity based on abundance class data—**c**), and their predictions globally at the whole area during the monitoring period (2003–2020)

The decrease in the beta diversity (based on presence/absence data), as well as its component taxonomical turnover, was observed between sites during the monitoring period (Fig. 3a, b). In beta diversity, it represented a 0.5% mean decline per year, whereas in the taxonomical turnover, it was a 0.7% mean decline per year. Based on abundance class data, the mean annual decrease in beta diversity value reached 0.6% (Fig. 3c).

**Fig. 4:**
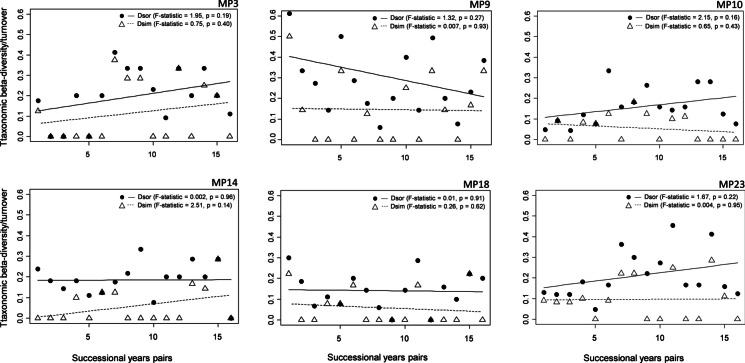
Change of the taxonomic beta diversity (Sorenson pairwise dissimilarity—Dsor), taxonomic turnover (Simpson pairwise dissimilarity—Dsim), and their predictions during the monitoring period (2003–2020). Explanation: black solid and dashed lines represent the predictions

In the particular monitoring sites, different trajectories were observed in the beta diversity and taxonomical turnover (Fig. 4). At the MP3, MP10, and MP23, a non-significant increase (at the significance level *ɑ* = 0.05) in beta diversity between successional years was determined, whereas a non-significant decrease was noted at the other three monitoring sites.

The non-significant increase in the taxonomical turnover was identified at the MP3 and MP14, whereas at the other sites, only a slight decline was indicated. Similar trends in temporal change of beta diversity based on incidence data were also observed in beta diversity based on the abundance class data, except MP23, where its significant increase was identified (Supplementary material Appendix Fig. 1).

### Macrophyte functional diversity and its change

From 2003 to 2020, functional richness decreased across all sites (except MP14) and the entire area, with a significant decrease (*p*-value < 0.05) observed at three sites as well as in the whole area (Table 2). At MP14, a relatively significant increase in both parameters evaluating functional alpha diversity was identified. In the case of functional dispersion, a significant decrease in values was observed at four sites. Based on the regression coefficient, the highest decline in functional richness and dispersion was found at the downstream sites MP18 and MP23, as well as at the upstream site MP3.

**Table 2:** Functional diversity of the macrophyte community and its prediction at each sampling site during the monitoring period (2003–2020)

Sites	Mean functional richness	Maximal functional richness	Minimal functional richness	Equation of trend line (within-site temporal change of functional richness)	Coefficient of determination *R*^2^ (%)	Randomisation test for regression coefficient (permuted *p*-value)
MP3	15.13	44.34	0.61	*y* = 4410 − 2.18 * year	53.7	0.002
MP9	12.32	22.12	0.21	*y* = 865.9 − 0.42 * year	6.8	0.323
MP10	37.35	69.32	1.36	*y* = 1444.9 − 0.69 * year	3.4	0.474
MP14	7.70	49.36	0.13	*y* = − 2219.8 + 1.11 * year	20.3	0.083
MP18	70.75	205.46	0.35	*y* = 12,779 − 6.32 * year	28.1	0.046
MP23	36.66	63.150	1.09	*y* = 5647.1 − 2.79 * year	47.4	0.005
Whole area	13.86	29.11	3.21	*y* = 5217 − 1.17 * year	52.4	0.003
Sites	Mean functional dispersion	Maximal functional dispersion	Minimal functional dispersion	Equation of trend line (within-site temporal change of functional dispersion)	Coefficient of determination *R*^2^ (%)	Randomisation test for regression coefficient (permuted *p*-value)
MP3	3.37	3.89	2.88	*y* = 60.89 − 0.03 * year	31.5	0.018
MP9	3.70	4.37	2.29	*y* = 22.27 − 0.009 * year	0.7	0.766
MP10	3.83	4.18	3.42	*y* = 55.29 − 0.03 * year	45.4	0.003
MP14	2.79	4.45	1.79	*y* = 190.34 + 0.09 * year	40.9	0.007
MP18	3.31	3.99	2.47	*y* = 46.75 − 0.02 * year	6.2	0.352
MP23	3.01	3.90	1.63	*y* = 103.22 − 0.05 * year	22.1	0.049
Whole area	3.46	3.75	2.79	*y* = 39.26 − 0.02 * year	14.8	0.137

**Fig. 5:**
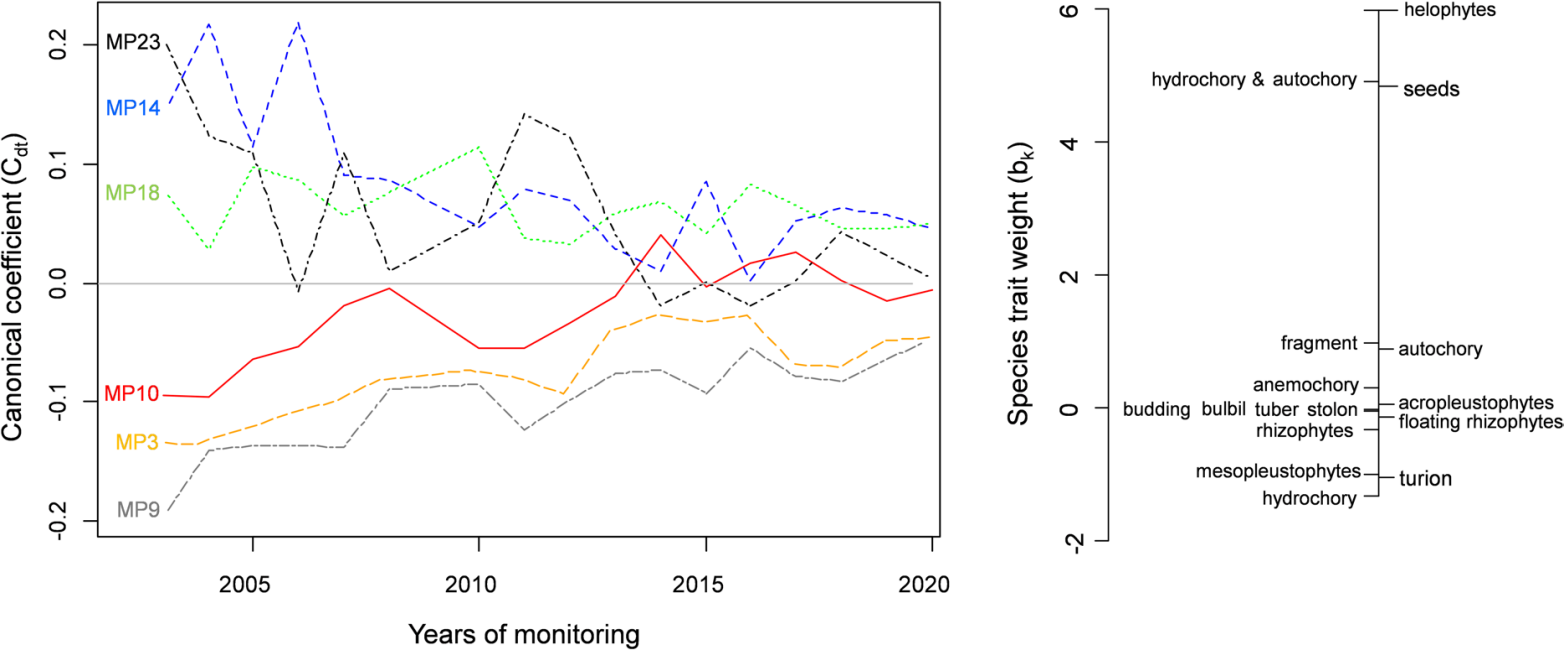
Principal response curves (PRC) showing temporal changes in the structure of the functional composition of macrophyte assemblages at the monitoring sites. The secondary axis (right) represents the first canonical axis of the Redundancy Analysis performed on the macrophyte functional traits weighed by species abundance classes

The PRC analysis revealed that 79.7% of the total variation in functional composition was due to within-site variation (including its interaction with the year), while 20.3% was attributed to between-year variation. The 53.9% of the total variation is represented by the first canonical axis of the PRC analysis. The PRC diagram also shows a clear decrease in the functional heterogeneity of the macrophyte community over the 20-year monitoring period (Fig. 5).

Similarly to PRC analysis based on taxonomical data, the multiple of b_k_ × C_dt_ determines the response pattern of each species trait proportion in the community over time. In the upstream sites (MP3–MP10), the most affected modalities within the analysed species traits were mesopleustophytes within the growth-form trait, hydrochory within the dispersal strategy, and turions spreading within dispersal units, all with negative weights, indicating a successional decline in their proportion during the monitored period. In the downstream sites (MP14–MP23), the most affected macrophyte modalities were helophytes within growth forms, hydrochory and autochory within the dispersal strategy, and seeds spreading within dispersal units. During the monitored period, their proportion in the functional composition of the community significantly decreased.

**Fig. 6:**
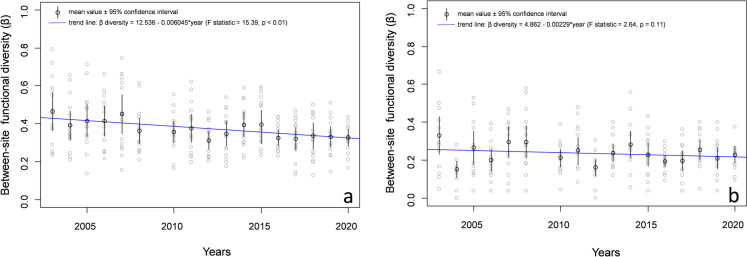
Change of the functional beta diversity and their predictions performed **a** on the species abundance classes (Bray—Curtis pairwise dissimilarity) and **b** the species incidence data (Sørensen pairwise dissimilarity) globally at the whole area during the monitoring period (2003–2020)

A decrease in functional beta diversity was found between sites during the monitoring period (Fig. 6a, b). Based on abundance class data, the mean annual decline represented 0.7%, while based on incidence data, it was approximately 0.3%.

**Fig. 7:**
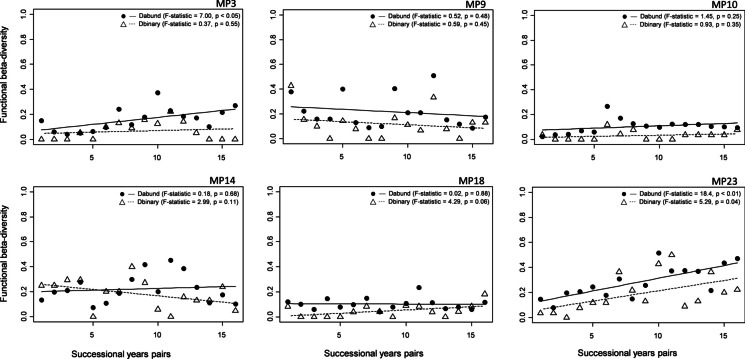
Change of the functional beta diversity based on the species abundance classes (Bray—Curtis pairwise dissimilarity) and species incidence data (Sørensen pairwise dissimilarity) and their predictions during the monitoring period (2003–2020). Explanation: black solid and dashed lines represent the predictions

In the particular monitoring sites, different trajectories were found in the values of functional beta diversity (Fig. 7). During the monitored period, a significant increase (at the significance level *ɑ* = 0.05) was noted at the MP3 (based on abundance class data) and MP23 (based on abundance class/incidence data). At the other monitoring sites, a non-significant increase/decrease in functional diversity of the macrophyte community was observed.

## Discussion

The results of our study illustrate that a relatively small area of the Danube Inland Delta represents a unique biotope from the view of macrophyte diversity. In comparison, the total species richness of 63 species (including 1 neophyte) found at six monitoring sites there is within the range of the findings of Dorotovičová and Oťaheľová (2008), Dorotovičová (2013), and Baláži and Hrivnák (2016), who reported between 43 and 102 species in artificial riverine canals across the Slovak Pannonian lowland. According to Hrivnák et al. (2013), the macrophyte species composition of the riverine canals is similar to natural water bodies, with stagnant or slowly flowing water in the Slovakian part of the Pannonian region. Results of the current macrophyte mapping of the entire Danube River show a similar number and composition of native species also in its main channel (Janauer et al., 2021). The Danube Delta in Romania represents a similar river landscape with similar environmental conditions, and according to several studies (e.g. Sârbu et al., 2011; Schneider-Binder, 2018), the taxonomic diversity of macrophytes is almost identical to the Danube Inland Delta. Despite many human influences, the study area provides suitable habitats for the existence of aquatic plants characteristic of the Danube basin.

The most frequent macrophyte taxa with high constancy of occurrence during the study period, including *C. demersum* (mesopleustophyte), *L. minor*, *S. polyrhiza* (acropleustophytes), and *C. acuta/gracilis*, *G. maxima*, *I. pseudacorus*, *P. australis*, *R. amphibia* (helophytes), are generally common and relatively widespread in various lowland aquatic habitats in Slovakia as well as throughout Central Europe (Chytrý, 2011; Dorotovičová, 2013; Dorotovičová & Oťaheľová, 2008; Janauer & Exler, 2004; Jursa & Oťaheľová, 2005; Szoszkiewicz et al., 2014). The three free-floating plant species (meso- and acropleustophytes) mentioned above are typical of their broad ecological tolerance towards habitat physical degradation, and so they can be considered extreme ecological generalists (Gebler & Szoszkiewicz, 2022). It is generally known that these taxa commonly inhabit moderately to highly eutrophic aquatic environments of the natural and hydromorphologically modified watercourses (Janauer & Wychera, 2000; O’Hare et al., 2006; Haury et al., 2006; Hachol et al., 2019; Szoszkiewicz et al., 2020). After the decline of water level in the main Danube channel caused by GHP operation, a decrease in the frequency of interconnection and flushing of habitats could create suitable conditions (e.g. higher trophy, higher summer temperature, stagnant water) for *C. demersum*, *L. minor*, and *S. polyrhiza*, as well as other pleustophytic plants (Szoszkiewicz et al., 2020; Van Dyck et al., 2021). Moreover, this fact can be supported by the high constancy of occurrence of some helophyte species (e.g. *G. maxima*, *P. australis*) as indicators of succession and eutrophication of the habitats (Hroudová & Zákravský, 1999; Tarkowska-Kukuryk et al., 2022; Čížková et al., 2023). Negative long-term changes induced by eutrophication, leading to simplification of community structure and dominance of species adapted to high nutrient content, e.g. *C. demersum*, have also been recorded in the Romanian Danube delta (Cristofor et al., 2003). On the other hand, recent findings indicate that even widely distributed aquatic plants may show a decrease in genetic diversity caused by changes in the water regime. According to Engloner et al., (2023, 2024), the genetic variability of *C. demersum* populations depends on hydrological connectivity and water flow, being lowest in isolated waters without flooding. This leads us to the assumption that the outcome of the biotic homogenisation of the aquatic environment can be the spread of hydrophytes with a wide ecological valence but with a reduced population genetic diversity.

In the Danube main channel, 4 neophytes were recorded: *Azolla filiculoides*,* Elodea canadensis*,* E. nuttallii*, and *Vallisneria spiralis* (Janauer et al., 2021)*.* Of these, *E. nuttallii* was the only invasive species detected at three (MP9, MP14, MP18) of the six monitored sites. While its presence was sporadic at the MP14, it occurred continually and in high quantity (25–75% site coverage) at the MP9 and MP18 until 2013. After 2013, the occurrence of *E. nuttallii* was not regular, and its quantity was significantly lower there (up to 5%). At the turn of the second millennium, several studies (e.g. Oťaheľová, 1996; Ohrádková, 1998; Oťaheľová & Valachovič, 2003) confirmed that the distribution of *E. nuttallii* had increased rapidly in the Danubian Lowland. Cao et al. (2023) documented the negative effect of floods on the biomass of *E. nuttallii* during the vegetation season. They also found that higher turbidity caused by flooding, together with the fast decomposition rate of *E. nuttallii*, can lead to a very slow recovery of this alien species. At both sites, a significant decrease in *E. nuttallii* biomass could be so explained by the extreme summer flood on the Danube in 2013.

The effect of *E. nuttallii* on macrophyte community diversity is not clearly negative. According to Bubíková et al. (2021), the presence of the species had only a weak effect on the alpha diversity of the communities but caused a significant decrease in beta diversity. Kelly et al. (2015) observed a positive effect on the alpha diversity of communities with the occurrence of the species. Zelnik et al. (2022) observed no impact on species diversity within communities. These three examples, among several studies, suggest that invasive aquatic plant species might not be the main drivers of biotic homogenisation in freshwater ecosystems.

Investigating the causes and consequences of biotic homogenisation/differentiation in aquatic environments is typically very challenging and has been addressed in only a few studies (e.g. Petsch, 2016; Price et al., 2018; Atanackovič et al., 2020), primarily focusing on natural aquatic assemblages. Moreover, homogenisation or differentiation is typically not a smooth or consistent process; it can be interrupted by periods of stability or even by the reverse process (Daga et al., 2015). This highlights the importance of using multiple time points in analyses to avoid misinterpreting broader trends based on short-term deviations (Clavero & Garcia-Berthou, 2006). In this context, the abiotic and biotic face of the Danube in the Bratislava region was significantly altered by the GHP. Its operation was mirrored in the change in the extensity and frequency of hydrological interconnections between riverine habitats. Our findings, resulting from long-term biotic monitoring, do not confirm the hypothesis that the loss of lateral connectivity, specifically in plesiopotamal channel types, leads to an increase in the species richness of macrophytes (Tockner et al., 1999). Analysing spatio-temporal dynamics of the aquatic macrophyte community, we found a successional decline in its taxonomical and functional heterogeneity. Our results can also support the statement that occasional flood disturbances are crucial, as they slow down vegetation succession, potentially preventing the decline in aquatic macrophyte species diversity (Bornette & Amoros, 1996).

Evidence of the homogenisation was found at the taxonomic and functional level of the studied community. In the German streams, the loss of the aquatic macrophyte species has also been associated with functional homogenisation of the community caused by the dominance of the species traits related to mechanical stress tolerance (Steffen et al., 2013). Some studies examining macrophyte changes in natural and human-influenced lakes (e.g. Lindholm et al., 2020) have identified the homogenisation of communities at the functional level but not at the taxonomical level. One possible explanation for this homogenisation pattern is the high intraspecific trait variation among aquatic macrophyte species, as many of them display considerable phenotypic plasticity in both their morphology and ecology. Due to intraspecific morphological and physiological plasticity, some species can exhibit different growth forms depending on the environmental conditions (Wetzel, 2001). According to Fu et al. (2014), the range of the intraspecific trait heterogeneity in macrophytes is a crucial factor in shaping community dynamics, and, in addition, it has also been found to contribute to functional beta diversity patterns (Spasojevic et al., 2016). In our case, however, the reduction in functional heterogeneity caused by the decline in the proportion of some traits related to growth form and dispersion strategy was associated with the loss of macrophyte species or a decrease in the abundance of some species.

While in the Danube Inland Delta, the environmental changes have mainly been caused by hydrological alterations, and in the North European lakes, environmental changes were primarily caused by shifts in water chemistry (Lindholm et al., 2020). The hydromorphological/hydrological characteristics of river habitats are usually considered more important than water chemistry for the occurrences of individual riverine macrophyte species, in particular under less extreme eutrophic conditions (Kaijser et al., 2022). Moreover, in recent decades, nutrient gradients have declined across much of Central Europe, with extreme nutrient pollution largely eliminated, although nutrient-poor sites remain rare. Under these conditions, the contributions of hydrological and hydromorphological impacts become indeed more relevant in macrophyte community shaping (Steffen et al., 2013). In the inland Danube delta, taxonomical and functional convergence of the aquatic plants had been continually ongoing over almost two monitored decades, and it can probably be attributed mainly to hydrological changes and natural succession of the area.

## Conclusion

This study provides insights into the taxonomical and functional changes within the aquatic macrophyte community in the middle stretch of the Danube River over nearly two decades. It is well established that river regulation and impoundment typically lead to the degradation of natural seasonal hydrological dynamics and a reduction in the lateral connectivity of riverine habitats. This is evident in the Danube River below Bratislava, where the unique aquatic habitat known as the “Danube Inland Delta” has been hydrologically disrupted by the construction and operation of the Gabčíkovo Waterworks. While we observed a decrease in both taxonomical and functional richness of macrophytes at most monitored sites, the expected successional increase in taxonomical and functional similarity of the assemblages was not confirmed. However, there was a clear trend toward taxonomical and functional homogenisation across the entire macrophyte community, likely reflecting environmental uniformisation of habitats throughout the Danube Inland Delta, significantly influenced by the operation of the Gabčíkovo Waterworks. Our findings show the loss or decrease in abundance of some plant species representing certain functional traits, e.g. helophytes, mesopleustophytes, and species with hydrochrous dispersal strategy. Given the crucial structural and functional roles that macrophytes play in aquatic environments, it is essential to consider measures to prevent further deterioration of their biodiversity. Therefore, these data may serve as an important foundation for assessing the effectiveness of planned actions aimed at restoring or improving the hydrological regime of habitats within the Danube Inland Delta.

## Supplementary Information

Below is the link to the electronic supplementary material.

ESM 1 (174 KB)

High Resolution Image (TIF 557 KB)

ESM 2 (DOCX 13.0 KB)

## Data Availability

The datasets are available upon reasonable request from the corresponding author.
