# Supplementary figures and images for: Aquatic macrophyte dynamics in the Danube Inland Delta over the past two decades: homogenisation or differentiation of taxonomic and functional community composition?
Source: Environ Monit Assess. 2025 Feb 27;197(3):332. doi: 10.1007/s10661-025-13777-1 (PMC11868360; doi:10.1007/s10661-025-13777-1)

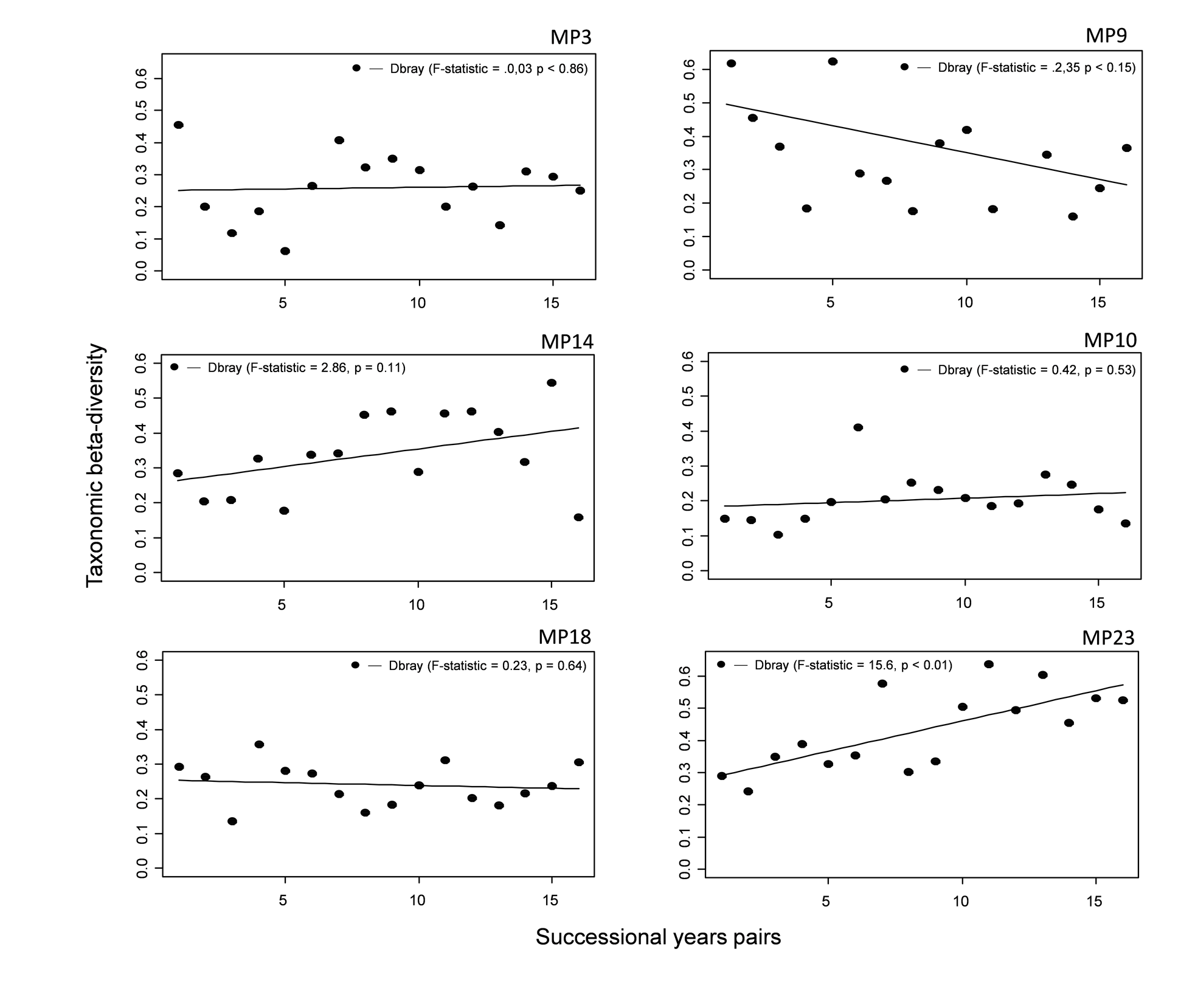

Supplement: Supplementary file 1 — (174 KB) [file 10661_2025_13777_Fig8_ESM.png]

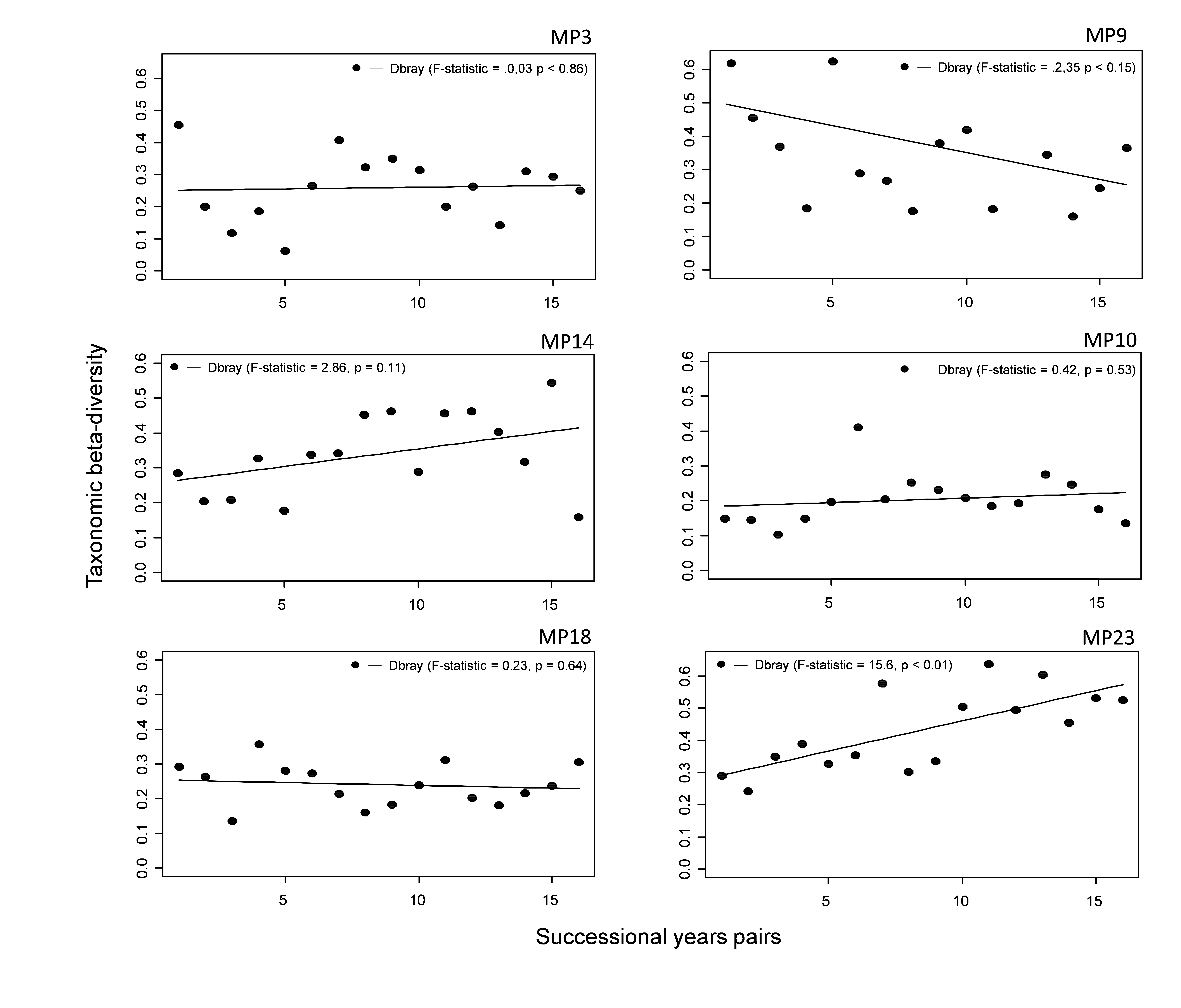

Supplement: Supplementary file 2 — High Resolution Image (TIF 557 KB) [file 10661_2025_13777_MOESM1_ESM.tif]
