# Supplementary material for: Aquatic macrophyte dynamics in the Danube Inland Delta over the past two decades: homogenisation or differentiation of taxonomic and functional community composition?
Source: Environ Monit Assess. 2025 Feb 27;197(3):332. doi: 10.1007/s10661-025-13777-1 (PMC11868360; doi:10.1007/s10661-025-13777-1)
Supplement: Supplementary file 3 — (DOCX 13.0 KB) [file 10661_2025_13777_MOESM2_ESM.docx]

Appendix Table 1 List of species traits used for the classification of macrophyte functional groups

| **Trait** | **Modality** | **Explanation** |
| --- | --- | --- |
|  | helophyte | emergent species of water (swamp) plants, rooting in the substrate |
|  | acropleustophyte | species floating on the water's surface and rooting freely in the water |
| Life form | mesopleustophyte | submerged species, non-rooting in the bottom substrate |
|  | rhizophyte | submerged species, rooting in the bottom substrate |
|  | floating rhizophyte | species with leaves floating on the water's surface, rooted in the bottom substrate |
|  | anemochory | dispersion by wind |
|  | hydrochory | dispersion by water |
| Dispersal strategy | autochory | dispersion without the aid of external agents |
|  | hydrochory & autochory | dispersion by water and also without the aid of external agents |
|  | seeds | seed dispersion |
|  | fragment | fragment dispersion |
|  | bulbil | dispersion by a secondary bulb that forms between leaf and stem |
| Dispersal unit | tuber | tuber dispersion |
|  | turion | dispersion by propagules that forms on the apical stems |
|  | stolon | dispesion by stems growing along bottom surface |
|  | budding | dispersion by budding |
